# Recommendations Provided to Families of Neurodivergent Children with Histories of Interpersonal Trauma across Two Clinical Assessment Services within a Major Metropolitan Children’s Hospital in Melbourne, Australia

**DOI:** 10.1007/s40653-024-00684-9

**Published:** 2025-01-28

**Authors:** Lauren A. Kalisch, Katherine A. Lawrence, Kelly Howard, Soumya Basu, Belinda Gargaro, Kypros Kypriano, Megan Spencer-Smith, Alexandra Ure

**Affiliations:** 1 School of Psychological Sciences, Monash University, Clayton, VIC 3800 Australia; 2 Early in Life Mental Health Services, Monash Health, Clayton, VIC 3168 Australia; 3 Developmental Paediatrics Unit, Monash Children’s Hospital, Clayton, VIC 3168 Australia; 4 Department of Paediatrics and Education Research, Monash University, Clayton, VIC 3168 Australia

**Keywords:** Interpersonal trauma, Autism, Intellectual disability, Intervention

## Abstract

Despite substantial evidence to suggest that neurodivergent children are particularly vulnerable to experiencing interpersonal trauma, evidence-based therapeutic guidelines for this group of children are lacking and best practice parameters have yet to be established. This study aimed to synthesise and describe recommendations provided by two specialist clinical assessment services within a major metropolitan children’s hospital in Melbourne, Australia, to families of children diagnosed with autism and/or an intellectual disability who have experienced interpersonal trauma. Twenty-six assessment reports written between 2021–2022 containing such recommendations were analysed. While less than half of the reports (43%) provided recommendations intended to directly support the child’s experience of interpersonal trauma, the majority (88%) provided ‘indirect’ recommendations that addressed other treatment targets known to benefit neurotypical children, or neurodivergent children without a history of trauma. Overall, these results shed light on the limited evidence-based strategies clinicians can reliably draw upon when working with this vulnerable population and reinforce the importance of developing empirically supported trauma interventions that are appropriate for neurodivergent children.

**Supplementary Information:**

The online version contains supplementary material available at 10.1007/s40653-024-00684-9.

Neurodivergent children, including those with autism spectrum disorder (autism) and/or an intellectual disability (ID), are at heightened risk of experiencing interpersonal trauma and its associated psychological consequences including depression and post-traumatic sequelae (Brenner et al., 2018; Jones et al., 2012; Mehtar & Mukaddes, 2011; Pfeffer, 2016; Reiter et al., 2007; Taylor & Gotham, 2016). This is likely due to a range of systemic factors that confer increased vulnerability (Algood et al., 2011) and intrinsic socio-cognitive and emotional processes that can amplify the risk of traumatisation (Gotham et al., 2014; Haruvi-Lamdan et al., 2020). Despite this evidence, the field currently lacks a clear roadmap of best intervention practices and key issues to consider when working with these children. Part of the challenge relates to there being insufficient evidence to reliably determine the appropriateness of existing trauma interventions for this population, and limited attempts to develop and assess the efficacy of novel therapeutic approaches (Kalisch et al., 2023). Therefore, it is of value to look towards a complementary source of knowledge that may offer guidance—namely, clinical expertise (Haggerty & Grace, 2008; Macnaughton, 1998; Parker, 2002).

Within the Australian public healthcare system, there are a number of services that offer comprehensive neurodevelopmental assessments to children, some of whom have a history of interpersonal trauma. As part of their assessments, clinicians within these services use their professional expertise to better understand children’s everyday functioning, experience of interpersonal trauma, developmental history, cognitive strengths and areas of difficulty, family circumstance, mental health status, and if applicable, their diagnostic profile (Goodall et al., 2023). At the conclusion of their assessment, clinicians provide families with a set of clear and actionable recommendations that are tailored to the individual characteristics of the child and their systemic context (Andren & Brown-Chidsey, 2012; Bornstein, 2017; Groth-Marnat & Wright, 2016; Harvey, 2006; Westervelt et al., 2007; Wright, 2020). As such recommendations represent a repository of clinical knowledge acquired within established practice context, their analysis may shed light on the types of trauma-informed programs, practices and intervention targets that clinicians prioritise in an effort to support neurodivergent children who have experienced trauma, as well as the specific recipients these practices intend to benefit (Andren & Brown-Chidsey, 2012; Groth-Marnat & Wright, 2016; Harvey, 2006; Westervelt et al., 2007; Wright, 2020).

This study aimed to synthesise and describe recommendations provided by two clinical assessment services within a major metropolitan children’s hospital in Melbourne Australia, to families of neurodivergent children who have experienced interpersonal trauma. This aim was achieved by exploring: (a) the demographic characteristics of children assessed; (b) to whom the recommendations were targeted (i.e., parent, school, wider community); (c) the types and frequencies of programs and psychosocial intervention targets that were recommended; and (d) the types and frequencies of trauma-informed psychosocial recommendations.

## Methods

### Sample

The sample comprised assessment reports written for families of children with a diagnosis of autism and/or an ID who experienced interpersonal trauma and underwent a comprehensive neurodevelopmental assessment between 2021 and 2022 at one of two participating clinical assessment services within a major metropolitan children’s hospital in Melbourne, Australia. The first was a medically specialised paediatric assessment service that focuses on complex neurodevelopmental presentations that may be impacted by additional psychosocial factors, such as interpersonal trauma. The service is comprised of psychologists, speech and language pathologists, and paediatricians. The second was a specialist psychiatric assessment team couched within a larger child and adolescent mental health service (CAMHS), made up of psychiatrists, psychologists, speech and language pathologists, and occupational therapists who address complex diagnostic questions related to whether an underlying neurodevelopmental disorder may be further complicating a young person’s psychiatric presentation.

Reports were included if they pertained to children aged 17 years or younger with a diagnosis of autism and/or an ID (including global developmental delay in children under five years of age) who had been exposed to interpersonal trauma, specifically: 1) physical, emotional, or sexual abuse, and/or 2) physical or emotional neglect, and/or 3) family violence. Reports were excluded if there was no identified diagnosis of autism and/or an ID, and/or reported exposure to interpersonal trauma.

### Clinical Assessment Processes

Between 2021–2022, children with autism and/or an ID and a history of interpersonal trauma underwent a comprehensive neurodevelopmental assessment at one of the two specialist clinical assessment services in Melbourne, Australia. Given that this period coincided with the COVID-19 pandemic where intermittent strict lockdowns were apparent, assessments frequently combined both Telehealth and in-person approaches. Whilst assessments varied based on the referral question, presenting issues, the child’s age and developmental level, they typically included: a detailed early life, medical and developmental history completed by parent/carer; a developmental or cognitive assessment; a medical and/or psychiatric assessment; administration of the Autism Diagnostic Observation Schedule (Lord et al., 2000); standardised parent questionnaires; allied health assessments (i.e., speech-language and/or occupational therapy); and a preschool/school visit if indicated. Assessment results were compiled into a report for the family, referrer, and other stakeholders, that contained information about the child’s demographic characteristics, clinical presentation, experience of trauma, diagnosis, formulation, and clinical recommendations.

### Screening and Data Extraction

A total of 223 reports of children seen in the clinics between 2021–2022 were de-identified and screened for eligibility. Due to the sensitive nature of the phrase ‘interpersonal trauma,’ a list of related terms was derived for screening purposes and included: complex background, challenging family history, difficult family environment, and disrupted attachment. In addition, reports that made references to alternative caregiving arrangements (i.e., foster care or guardianship arrangements), and/or specified the involvement of social services with known links to interpersonal trauma crisis support were included. This study was approved by the Major Metropolitan Children’s Hospital’s Human Research Ethics Committee (HREC) 89699. As this was a retrospective study that analysed de-identified reports, consent was not required.

Study data were extracted and managed using Microsoft Excel and the Research Electronic Data Capture Tool (REDCap; Harris et al., 2009). REDCap is a secure, web-based application that is hosted and managed by Helix (Monash University) to support data capture for research studies. An extraction template was developed and tested independently by two researchers (L.K & A.U) who extracted the relevant data from each report. Consensus meetings were held to explore extraction consistency and calibrate responses. In cases of inconsistency, verbal discussion and re-extraction were jointly undertaken until consensus was met.

Descriptive statistics and frequency calculations were used to report the demographic characteristics of the sample, while the recommendations provided to families underwent a summative content analysis. The summative content analysis involved identifying relevant and reoccurring passages of text, keywords, and/or other data to help differentiate important from unimportant information (Corbin & Strauss, 2008; Rapport, 2010; Strauss & Corbin, 1998). This process was used to categorise psychosocial recommendations into key themes (Fereday & Muir-Cochrane, 2006). Themes identified in a recent scoping review conducted by this research team were used to support the analysis (Kalisch et al., 2023), yet themes were iterated and updated as required.

Each theme pertained to a different therapeutic target (i.e., the core objective/focus of a therapeutic intervention). At the child level, therapeutic targets included: social communication; receptive and/or expressive language; emotional/psychological functioning (which included sensory processing given its association with children’s emotional regulation and functioning; (Brindle et al., 2015) interpersonal trauma/attachment difficulties; and capacity building, community participation and independence.

At the parent/caregiver level (hereafter referred to as ‘parent level’) targets included parent as client (i.e., whereby the intervention centred on parents receiving their own support), and parenting skills (i.e., whereby the intervention focused on upskilling parents with specific parenting strategies). At the educational level, cognitive/academic functioning was the only target included.

The frequency with which each target emerged across different age groups was recorded, along with relevant qualitative information. Explicit mention of any therapeutic programs (i.e., Zones of Regulation®; Kuypers, 2013) and/or modalities (i.e., cognitive behavioural therapy) was also recorded, along with any third party or external agency recommendations. Third party or external agency recommendations were reported descriptively, while other recommendations were presented graphically and in tabular form. As this study was interested in psychosocial recommendations, recommendations pertaining to pharmacological interventions were not included.

## Results

**Fig. 1:**
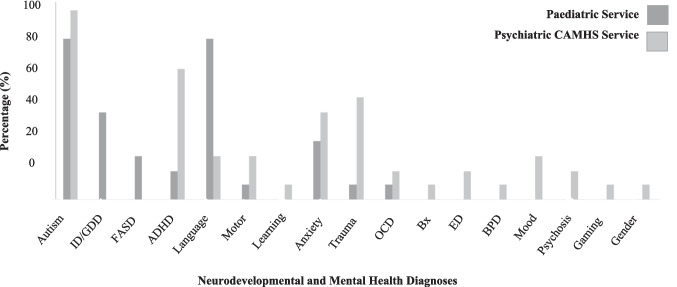
Percentage of children with neurodevelopmental and psychiatric diagnoses across services*.* Autism Spectrum Disorder (Autism); Intellectual Disability or Global Developmental Delay (ID/GDD); At Risk of Foetal Alcohol Spectrum Disorder (FASD); Attention Deficit Hyperactivity Disorder (ADHD); Language Disorder (Language); Developmental Coordination Disorder and/or other Motor Disorders (Motor); Specific Learning Disability (Learning); Anxiety Disorder (Anx); Trauma-related disorders including Complex Post Traumatic Stress Disorder and Post-Traumatic Stress Disorder (Trauma); Obsessive Compulsive Disorder (OCD); Severe Behaviour Disorder (Behaviour); Borderline Personality Disorder (BPD); Mood-related disorders including Depression (Mood); Gaming Disorder (Game); Gender Dysphoria (Gender)

Twenty-six assessment reports were identified that met the inclusion criteria. Table 1 and Fig. 1 display the demographic characteristics of the children assessed across services. Overall, children were aged between 1 and 16 years, wherein the cohort of children assessed within the paediatric service were younger in age. All children with an intellectual disability or global developmental delay were from the paediatric service (*n* = 6/26; 23%). The majority of children had two or more co-occurring neurodevelopmental diagnoses (*n* = 23/26; 88%), with many of these children having undergone an assessment within the paediatric service (*n* = 12/23; 60%). One child from the paediatric service had four co-occurring neurodevelopmental conditions (8%). Most children (*n* = 17/26; 65%) had a mental health diagnosis, and nearly half of these children had a specific trauma-related diagnosis (*n* = 8/17; 47%). A notable proportion of children had two or more co-occurring mental health conditions (*n* = 8/26; 31%), and it is worth acknowledging that all of these children were from the psychiatric CAMHS service.

**Table 1:** Demographic characteristics of children assessed across services

	Paediatric Service (*n* = 13)	Psychiatric CAMHS Service (*n* = 13)	Overall Sample (*n* = 26)
Child Age in years *M (SD)*	5.7 (2.8)	13.35 (3.0)	9.50 (4.8)
Gender *n* (%)			
Male	9 (69%)	5 (38%)	14 (54%)
Female	4 (31%)	6 (46%)	10 (38%)
Non-binary	0	2 (15%)	2 (8%)
Linked with an existing service *n (%)*	5 (39%)	12 (46%)	17 (65%)

Just under half the reports (*n* = 12/26; 46%) explicitly named the type of interpersonal trauma children had experienced which included exposure to domestic violence, emotional, psychological, physical and/or sexual abuse, and neglect. Contrastingly, the remaining reports (*n* = 14/26; 54%) provided less descript details of interpersonal trauma exposure, referenced alternative caregiving arrangements, and/or cited social services with known links to interpersonal trauma crisis support (see Supporting Information for full list of terminology used throughout the reports). Notably, most children (*n* = 17/26; 65%) who presented for assessment were already receiving some form of psychosocial support, which included support from a psychologist, psychiatrist, trauma counsellor, play therapist, and/or child protection worker.

### Child Level Recommendations (see Fig. 2 & Table 2)

**Fig. 2:**
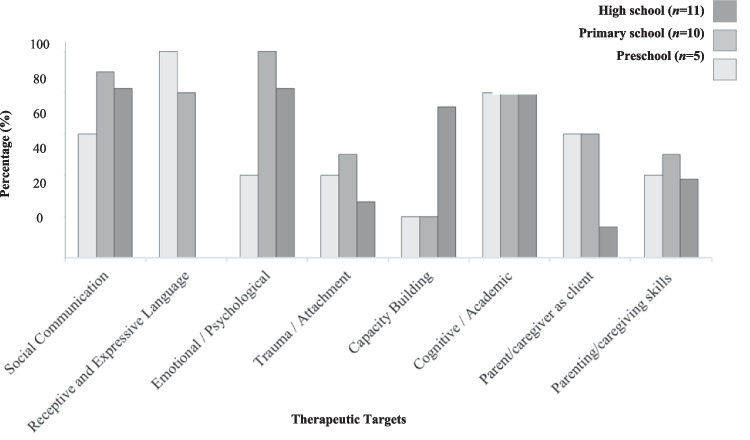
Percentage of therapeutic targets recommended across age groups

**Table 2:** Therapeutic targets recommended across reports differentiated by age group

Theme	Target	All Ages	Preschool Specific	Primary School Specific	High School Specific
Social communication	**Social pragmatics and skills**	• Support child to understand others’ non-verbal communication	• Use social stories	• Use social stories • Social Thinking® program	• Social Thinking® program • PEERS® program • Don’t force eye contact
	**Peer contact and relationships**	• Provide frequent opportunities for children to socialise and practice skills across different settings with peers of different ages • Social skills groups			• Support child to build social confidence • Employ a support worker to help child navigate relationships
	**Social communication style**	• Avoid using sarcastic, ambiguous, indirect, and/or metaphorical language			
Receptive and/or expressive language	**Communication aids and tools**		• Hanen More than Words® program • Picture Exchange Communication System ® • Key Word Sign • Use object symbols and visuals	• Use visuals	
	**Language processing**		• Use short sentences • Use less mature language • Ask open ended questions	• Use short sentences • Use less mature language • Ask open ended questions • Use written instructions • Contextualise information • Ask child to repeat things in their own words	
Emotional psychological	**Emotional literacy and regulation**	• Play music • Provide fidget tools • Teach child about feelings • Develop a sensory profile • Promote interoceptive awareness • Provide child with a safe, calm down space (i.e., with reduced lighting, bean bags, pillows, heavy blankets, a weighted lap pad, ear defenders, Lego) • Support mindfulness • Promote compression clothing • Support child to engage in heavy work activities	• Engage with child about their unique interests	• Ensure child is in close proximity to someone familiar • Zones of Regulation®	• Reduce screen time, • Zones of Regulation® • Support child to re-engage in pre-loved activities and activities of interest • Increase child’s self-esteem • Increase positive interactions • Promote autonomy and collaboration using the Collaborative and Proactive Solutions® framework • Help child engage in graded exposure exercises
	**Transitions, routine, and predictability**	• Allow adequate time for child to adjust to and learn new routines	• Use first, then instructions • Use visual schedules • Use social stories	• Name unfamiliar objects • Use visual schedules • Use social stories	
Trauma attachment	**General trauma and attachment support**	• Promote a safe and consistent caregiving environment for the child • Utilise trauma and family violence support services • Provide trauma informed psychology to the family		• Play therapy	• Trauma-focused cognitive behavioural therapy • Dialectical behaviour therapy • View behaviour through a trauma, relational and neurodiversity lens • Consider the child’s unmet needs that may be driving behaviour • Promote autonomy and collaboration using the Collaborative and Proactive Solutions® framework • Increase positive interactions with child
Capacity Building, Community Participation, Independence	**Independence and participation**	• Support personal care skills • Promote self-help skills • Support participation in community activities			• Employ a support worker to help child build confidence, and foster a sense of community connection • Promote engagement in meaningful areas of interest • Support employment training and provide career advice • Support living skills
Cognitive academic	**Classroom engagement**	• Consider the child’s unmet needs that may be driving behaviour • Minimise distractions • Speak slowly and clearly • Pause and give time for child to process instructions • Provide movement breaks and physical activity outlets • Minimise distractions • Use repetition • Give short instructions • Help child to organise their workspace • Use visuals • Establish routine at school		• Support child to sit at the front of the class	• Provide child with a mentor • Provide forewarnings • Reduce child’s stress • Provide a calming space • Use positive reinforcement • Reduce screen time • Take a nurturing and curious approach with child rather than using behavioural strategies
	**Learning support and curriculum management**	• Develop an individualised learning program • Modify the curriculum		• Reduced child’s workload • Reduce task complexity	
	**Collaboration and attendance**	• Arrange regular meetings with family • Communicate diagnosis to school • Arrange student support meetings • Pick the right school to match child’s needs			
Parent as client		• Engage in regular self-care • Establish regular opportunities for respite • Seek psychological support			
Parenting skills	**General strategies**	• Work together with a therapist to set functional goals as a family • Provide simple strategies that can be incorporated into the family’s daily life • Understand child’s distress cues • Empathise with child • Model self-regulation • Engage in slow deep breathing • Develop regulation strategies together			• Provide consistent responses to child’s behaviour • Use specific language to help child understand their feelings • Find compromises with child • Use strengths-based language • Make times for fun and laughter • Separate how the child feels from who the child is to minimise shame • Help child have a sense of choice and control over their environment • Notice feelings in a non-judgmental manner

^a^An empty box does not denote that age-specific recommendations are irrelevant or inappropriate. Rather, it suggests that age-specific strategies were not explicitly recommended across the analysed reports

#### Interpersonal Trauma/Attachment Difficulties

Almost all reports (*n* = 22/26; 85%), comprehensively described and/or acknowledged the experience of interpersonal trauma and its pervasive impacts on the child, while others briefly mentioned or alluded to the child’s experience of trauma in the background section (*n* = 4/26; 15%). Overall, less than half of the reports (*n* = 10/26; 38%) made a trauma-informed psychosocial recommendation. Recommendations ranged from promoting a safe and consistent caregiving environment, to repairing relationships with parents through empathy, attunement, and responsiveness, to receiving help from trauma-related family services, and receiving trauma-informed psychological interventions. Trauma-informed interventions that were recommended included play therapy, trauma-focused cognitive behavioural therapy (TF-CBT; Cohen et al., 2006), and dialectical-behavioural therapy (DBT; Linehan, 1993). For children who presented with behavioural challenges, it was recommended that such behaviour be viewed through a trauma, relational, and neurodiversity lens, with due consideration of the unmet needs that may be driving the child’s behaviour.

#### Social Communication

Most reports (*n* = 21/26; 81%) made a social communication recommendation. Recommendations were made across all age groups and centred on supporting children to establish meaningful connections with their peers, and improving others’ ability to understand neurodivergent communication styles and preferences (e.g., by minimising the use of sarcasm and non-literal language). The use of social stories was recommended to support social communication with younger children, while the Social Thinking® (Winner, 2008) and PEERS Program® (Laugeson, 2014) were recommended for older children.

#### Receptive and/or Expressive Language

Half of the reports (*n* = 13/26; 50%) recommended receptive and/or expressive language as a key therapeutic priority. While this therapeutic target was recommended for all preschool aged children, and most primary school aged children (*n* = 8/10; 80%), none of the high school aged children were offered a recommendation of this nature. Reports recommended supporting children’s receptive and expressive language skills by modifying communication (e.g., using shorter sentences, and asking open ended questions), and utilising communication aids (e.g., visual supports). In particular, for younger children who were minimally verbal, programs recommended for receptive and/or expressive language included: Hanen More than Words® (Sussman et al., 1999), Picture Exchange Communication System® (PECS; (Bondy & Lori, 1998), and Key Word Sign (KWS).

#### Emotional/Psychological Functioning

The majority of reports (*n* = 21/26; 81%) made a recommendation that centred on supporting children’s emotional/psychological functioning. Notably, while all primary, and nearly all high school (*n* = 9/11; 82%) age children were offered a recommendation in this category, this was not the case for preschool age children (*n* = 2/5; 40%). That said, consistent recommendations included strengthening children’s emotional literacy and interoceptive awareness, and supporting emotional regulation through, for example, music, social stories, fidget tools, physical activity, repetitious movement, mindfulness, and meditation. All reports suggested that children be supported with transitions, and providing routine and sensory outlets were considered integral to children’s emotional regulation. For older children specifically, it was suggested that re-engaging in pleasurable activities, increasing self-esteem, facilitating positive interactions, and minimising avoidance were paramount. The Zones of Regulation® program (Kuypers, 2013) was also recommended for the older age groups.

#### Capacity Building, Community Participation And Independence

Less than half of the reports (*n* = 11/26; 42%) suggested capacity building, community participation and independence as an intervention priority, however the majority of these recommendations were directed toward high school age children (*n* = 8/11; 71%). Therapeutic targets that spanned all age groups included supporting children with their personal care and self-help skills, and promoting engagement in community activities. For older children specifically, there was a focus on employment, career advice, and living skills.

### School/Educational Level Recommendations

#### Cognitive/Academic Functioning

A considerable portion of reports (*n* = 20/26; 77%) made an ‘educationally focused’ recommendation that centred on children’s cognitive and/or academic functioning. Such recommendations were evenly spread across age groups, and typically involved: providing learning support and curriculum management (i.e., developing individualised learning plans), enhancing classroom engagement and participation (i.e., by offering breaks and distraction aids, scaffolding tasks, using forewarnings and positive reinforcement), and supporting attendance and collaboration by establishing partnerships between parents and educational providers.

### Parent Level Recommendations

#### Parent as Client and Parenting Skills

A number of reports (*n* = 11/26; 43%) suggested that parents’ own mental health be the focus of intervention efforts, whereby respite, self-care, and psychological support should be prioritised. Separately, nearly half of the reports (*n* = 12/26; 47%) encouraged parents to develop specific parenting skills, including learning how to attune to a child’s distress cues, model self-regulation, engage in co-regulation and emotional mirroring, use strengths-based language, and increase ‘fun’ within the parent–child dynamic. It was also recommended that parents show empathy, understanding and curiosity with regard to children’s behavioural challenges, and support children’s autonomy and skill building using the Collaborative and Proactive Solutions® framework (Greene, 1998). Of note, nearly all reports (*n* = 22/26; 85%) offered families additional psychoeducational resources for them to review in their own time. Resources included website links, workshops, helplines, videos, podcasts, and book recommendations.

### Wider Level Recommendations

#### Third Party/External Agency

All reports recommended that families receive funding support (see Table 1). The most common funding option was the ‘National Disability Insurance Scheme’ (*n* = 25/26 reports; 96%), which provides funding to support people with a disability and their families or carers who hold permanent residency or citizenship within Australia. Nearly all reports (*n* = 23/26; 88%) recommended ongoing allied health involvement as a support priority for children. Recommended practitioners included speech pathologists, occupational therapists, psychologists, and play therapists. It was also recommended that some children (*n* = 15/26; 58%) engage with specialist clinical and/or social services that offer welfare, general wellbeing, and psychological support. Services included Child Adolescent Mental Health Services, Child Protection Services; Autism Information Services; Youth Support Services, and in-patient admissions to local hospitals in Melbourne, Australia.

## Discussion

The existing empirical literature offers little guidance on how to best support neurodivergent children who have experienced interpersonal trauma. Therefore, we looked toward clinical expertise, a complimentary source of evidence, and analysed recommendations provided to our population of interest by clinicians practicing in a major metropolitan children’s hospital. Frequently described as “the primary purpose of an assessment” (Wright, 2020), such recommendations offered possible ways to improve the mental health, general functioning, and overall quality of life of these vulnerable children and their families.

There was considerable variability in the characteristics of children assessed, whereby children differed in age, reported experience of interpersonal trauma, gender, neurodevelopmental diagnosis(es), and psychological profile. Results revealed that younger children with more complex neurodevelopmental presentations were assessed within the paediatric service (i.e., up to four neurodevelopmental diagnoses), while the psychiatric CAMHS service was marked by an older cohort of children, with stronger language and cognitive abilities, and greater mental health complexity (i.e., up to five co-occurring mental health disorders). Indeed, it is well established that the onset of many mental health disorders occurs in adolescence, and that mental health comorbidity typically increases with age (Australian Institute of Health & Welfare, 2016; Bitsko et al., 2022; Ghandour et al., 2019; Solmi et al., 2021). However, the relationship between a child’s age at diagnosis, their neurodivergent characteristics, and mental health status cannot be overlooked when interpreting these results. Specifically, evidence suggests that children with more ‘apparent’ neurodevelopmental differences tend to be diagnosed at an earlier age, and subsequently gain increased access to timely intervention and support (Hosozawa et al., 2021; Rosenberg et al., 2011; Sheldrick et al., 2017). Comparatively, for children with less ‘observable’ features, like in the psychiatric CAMHS service, their difficulties often go undetected in childhood, and they consequently bear a heavy burden of mental health challenges due in part to the delay in diagnosis (Hosozawa et al., 2021; Livingston & Happé, 2017; Mandy et al., 2022; Mazurek et al., 2014; Zwaigenbaum et al., 2019). It is therefore possible that the psychiatric complexity inherent to those assessed within the CAMHS service was not simply a biproduct of adolescence being marked by increased psychologically vulnerability, but rather a consequence of their neurodiversity being historically overlooked. Ultimately, the results suggest that the ‘right’ children navigated their way to the ‘right’ assessment service, and whilst the circumference of both services spans both neurodiversity and mental health domains, the separate streams confer the added benefit of specialisation and expertise.

Our results further identified that recommendations targeted children, their parents, the educational setting, and the wider service systems that sit around the child (i.e., external agencies, social services, and funding bodies). At the parent level specifically, there was a distinct focus on parents’ mental health as a valuable support priority in its own right. This is consistent with research on the importance of specifically addressing parental stress and mental illness in interpersonal trauma prevention and re-occurrence (Algood et al., 2011; Bagur et al., 2022; Black et al., 2001; Cicchetti & Lynch, 1993; Rodriguez et al., 2019). Another key theme to emerge was that parents should be supported with recognising and managing their own emotions, and supportively responding to their children’s emotions. These processes, collectively referred to as ‘parental emotional socialisation’ or ‘emotion-focused’ parenting have been identified as crucial to fostering healthy parent–child relationships and optimising children’s emotional development (Camoirano, 2017; Eisenberg et al., 1998; England-Mason & Gonzalez, 2020; Hajal & Paley, 2020). Overall, these ecologically informed findings reinforce the importance of casting a wide net of support around the child that extends well beyond the therapy room (Algood et al., 2011; Bronfenbrenner, 1986).

We also set out to explore the types and frequencies of psychosocial programs and intervention targets that were recommended. Here we discovered that ‘emotional/psychological functioning’ and ‘social communication’ accumulated the highest number of recommendations across age groups respectively. Admittedly, these intervention domains are routinely endorsed for neurodivergent children who have *not* experienced trauma, nevertheless, their significance may be even more pronounced when trauma is a factor. Specifically, difficulties with emotional regulation are a well-documented outcome of trauma (Dvir et al., 2014), particularly for neurodivergent children due to the added strain on an already overwhelmed emotion regulation system (Haruvi-Lamdan et al., 2018; Jones et al., 2012; Kerns et al., 2015; Mazefsky et al., 2013). Further, studies have demonstrated that interpersonal relationships and peer interactions are protective in facilitating trauma recovery (Calhoun et al., 2022; Fredette et al., 2016). Therefore, enhancing others’ understanding of neurodivergent social-communication styles, and providing support to neurodivergent children who wish to strengthen their relationships with peers may serve to mitigate some of the adverse impacts of trauma. Taken together, these results shed light on potential evidence-based intervention targets that may have the capacity to *indirectly* mitigate the effects of trauma in neurodivergent children (Paintain & Cassidy, 2018; Reddemann & Piedfort-Marin, 2017).

An additional finding was that for preschool age children in particular, improving ‘receptive and/or expressive language’ was a recurrent recommendation, however there was less emphasis on supporting emotional/psychological functioning and/or interpersonal trauma/attachment difficulties in this age group. This may be due, at least in part, to the high incidence of language difficulties among children assessed within this age bracket. Nevertheless, it is also possible that clinician’s purposefully prioritised foundational language skills ahead of explicit mental health or trauma strategies, given the fundamental role that language plays in children being able to process and express their emotional experiences (Lindquist et al., 2015). An alternative explanation is that the emotional/psychological impacts of trauma may have been overshadowed by the pervasiveness of these children’s language difficulties, and therefore became a primary focal point (Reiss et al., 1982). Overall, it appears as though age and language level had some bearing on the nature, type and frequency of recommendations provided, and should be considered as key variables to explore further in future studies.

Perhaps the most important discovery, was that less than half the reports made a recommendation targeting ‘interpersonal trauma/attachment difficulties’ directly. Indeed, at the time of assessment, a number of children were already receiving some form of psychosocial support. As such, clinicians may have believed that such supports were already trauma-informed, and therefore believed it unnecessary to explicitly list specific trauma interventions in their recommendations. Nevertheless*,* as reports were distributed to multiple stakeholders involved in the child’s care, it is reasonable to assume that clinicians would have capitalised on the opportunity to formally document and recommend a trauma-informed intervention, irrespective of whether a child had already been linked in with a support. Of the few reports that did provide a ‘direct’ recommendation, three, evidence-based trauma interventions were listed (i.e., TF-CBT, DBT and play therapy; Baggerly et al., 2010; Cohen et al., 2006; Linehan, 1993). While these interventions have yet to be robustly evaluated with neurodivergent children who have experienced interpersonal trauma, a growing body of literature demonstrates their effectiveness in reducing the impacts of trauma within the general child population (Baggerly et al., 2010; Bratton et al., 2005; Cohen et al., 2004; Geddes et al., 2013; Humble et al., 2019). Furthermore, consistent with decades of research on attachment theory, a number of reports underscored empathy, attunement, and responsiveness as being integral to optimising children’s well-being, cultivating secure parent–child relationships, and promoting safety, stability, and connection in the wake of interpersonal trauma (Bowlby, 1982, 1988; Cicchetti & Lynch, 1993; Hoffman et al., 2006; Lieberman et al., 2019). Although only three evidence-based interventions were explicitly mentioned, it is encouraging that these ‘core attachment ingredients’ which are foundational to an array of evidence-based attachment therapies emerged across reports.

On balance, it was clear that clinicians acknowledged children’s experience of interpersonal trauma and its impact. Overall, however, there was often a lack of specificity on how to explicitly support children and their families cope with interpersonal trauma and its sequelae. This here reflects the great divide between neurodiversity and trauma research that has pervaded the literature, and the reality that intervention efforts across these disciplines have yet to be effectively consolidated in the child population (Kalisch et al., 2023). Indeed, while the two assessment teams in this study sit at the forefront of integrated neurodiversity and trauma clinical services, the research sector has only recently begun to welcome this juncture (Dodds, 2020; Fuld, 2018; Kerns et al., 2015; Kildahl et al., 2019; Peterson et al., 2019; Rumball, 2019; Stack & Lucyshyn, 2019). In the absence of evidence to guide recommendations, it is therefore unsurprising that clinicians relied on targets and strategies that did not always obviously target the trauma, but did have a substantial evidence base behind them. To advance this field, future studies will need to invest in intervention research at the nexus of trauma and neurodiversity, and clarify whether novel trauma interventions are needed for this population, and/or whether existing therapies can be modified to meet the needs of neurodivergent children. Additionally, future studies should consider trying to understand how and why clinicians select particular recommendations for individual children and families.

Whilst this study makes a meaningful step toward supporting these vulnerable children and their families, it is not without limitations. It is known that the most effective assessment reports are those that are sensitive to their intended readership (Postal & Armstrong, 2013). As reports were written with families in mind, as well as educators with whom families may not have wanted their private and sensitive information shared, it is possible that clinicians chose not to overt the trauma, and/or magnify its visibility within the recommendations. This may, in part, account for the limited number of recommendations in the interpersonal trauma/attachment difficulties domain. However, it is also plausible that interpersonal trauma/attachment difficulties were not solely targeted via the provision of specific recommendations, but may have also been addressed through sharing a comprehensive trauma formulation with families. While this study aimed to synthesise and describe ‘explicit’ therapeutic recommendations, future research may consider the power of the formulation as a therapeutic tool in its own right (Ruggiero et al., 2021). It is also important to acknowledge that in addition to age and language ability, different characteristics such as neurodevelopmental diagnosis, level of intellectual ability, degree of independence and support needs, mental health difficulties, type of trauma exposure, living arrangements, and family structure, hold the potential to impact any therapeutic recommendations provided. Whilst the sample size of this study did not allow for statistical comparisons to be made across recommendations based on these factors, future research should consider how to better differentiate what works for which types of children through multicentre studies. Further, research should also aim to clarify whether the recommendations provided at assessment are ultimately taken up by families as they navigate the ‘intervention landscape,’ or whether barriers at the child, family or treatment provider level limit the extent to which recommendations are enacted.

## Conclusions

This study was the first of its kind to analyse clinical recommendations provided by a cross section of specialist assessment services that work with neurodivergent children who have experienced interpersonal trauma in a major metropolitan children’s hospital. The findings highlight the importance of creating a network of supports at the parent/caregiver, school, and wider community levels around the child, and suggest that intervention should be viewed as a systemic phenomenon that extends beyond the bounds of a conventional therapeutic setting. In addition, the clinical expertise gleaned from these services revealed several promising strategies that differed slightly across age groups. These strategies addressed interpersonal trauma directly (i.e., were visibly trauma-informed), or indirectly via other treatment targets that have been independently recognised as effective for both neurodivergent children who have not experienced trauma, and neurotypical children who have. Overall, our results speak to the reality of siloed neurodiversity and trauma research efforts, whereby there is now a paucity of evidence-based, trauma-informed interventions for even skilled clinicians to draw upon when offering therapeutic recommendations to neurodivergent children. As such, it is incumbent upon future studies to make this research area a priority and develop evidence-based trauma interventions for this vulnerable population.

## Supplementary Information

Below is the link to the electronic supplementary material.

Supplementary file1 (DOCX 16 KB)

## Data Availability

The de-identified reports analysed during the current study will not be available in a public repository as ethics approval was not obtained for this purpose.
