# Supplementary material for: Recommendations Provided to Families of Neurodivergent Children with Histories of Interpersonal Trauma across Two Clinical Assessment Services within a Major Metropolitan Children’s Hospital in Melbourne, Australia
Source: J Child Adolesc Trauma. 2025 Jan 28;18(2):467–80. doi: 10.1007/s40653-024-00684-9 (PMC12130377; doi:10.1007/s40653-024-00684-9)
Supplement: Supplementary file 1 — Supplementary file1 (DOCX 16 KB) [file 40653_2024_684_MOESM1_ESM.docx]

Supporting Information

**Full List of Terminology used across reports to describe interpersonal trauma.**

Early childhood trauma; complex social background with child protection involvement; complex psychosocial history; difficult family environment; family history of trauma; core underlying difficulties related to attachment dynamics; interpersonal difficulties within the family; disrupted attachment during early life; exposure to traumatic experiences; considerable stress in the family environment; background of significant trauma; complex family and environmental stressors; unpredictable and inconsistent care; adverse childhood experiences; and early childhood complexities, traumas and tragedies.
